# Cars, corporations, and commodities: Consequences for the social determinants of health

**DOI:** 10.1186/1742-7622-5-4

**Published:** 2008-02-21

**Authors:** James Woodcock, Rachel Aldred

**Affiliations:** 1 Department of Epidemiology and Population Health, London School of Hygiene and Tropical Medicine, London, UK; 2 University of East London, School of Social Sciences and Media Studies, London, UK

## Abstract

Social epidemiologists have drawn attention to health inequalities as avoidable and inequitable, encouraging thinking beyond proximal risk factors to the causes of the causes. However, key debates remain unresolved including the contribution of material and psychosocial pathways to health inequalities. Tools to operationalise social factors have not developed in tandem with conceptual frameworks, and research has often remained focused on the disadvantaged rather than on forces shaping population health across the distribution. Using the example of transport, we argue that closer attention to social processes (capital accumulation and motorisation) and social forms (commodity, corporation, and car) offers a way forward. Corporations tied to the car, primarily oil and vehicle manufacturers, are central to the world economy. Key drivers in establishing this hegemony are the threat of violence from motor vehicles and the creation of distance through the restructuring of place. Transport matters for epidemiology because the growth of mass car ownership is environmentally unsustainable and affects population health through a myriad of pathways. Starting from social forms and processes, rather than their embodiment as individual health outcomes and inequalities, makes visible connections between road traffic injuries, obesity, climate change, underdevelopment of oil producing countries, and the huge opportunity cost of the car economy. Methodological implications include a movement-based understanding of how place affects health and a process-orientated integration of material and psychosocial explanations that, while materially based, contests assumptions of automatic benefits from economic growth. Finally, we identify car and oil corporations as anti-health forces and suggest collaboration with them creates conflicts of interest.

## Introduction

In this paper, we explain how thinking about social forms and social processes can develop epidemiological theory about the social determinants of health. We present the case of the car, the related social forms of the commodity and corporation, and the social processes of motorisation and capital accumulation. These concepts help to elucidate the car's role in production and consumption, and how this impacts on health. Our focus on these social forms and social processes introduces an area missing from social epidemiological work with implications for theoretical and empirical work. We discuss these implications for debates on psychosocial versus material pathways and the impact of place on health, before offering suggestions on how to progress the research agenda and identify major anti-health forces.

## Analysis

### Challenges facing social epidemiology

Health inequalities and the social determinants of health have become part of mainstream health discourse. In March 2005, the World Health Organization (WHO) set up a Commission on Social Determinants of Health [1] involving some leading global social epidemiologists.

Social epidemiologists have drawn attention to health inequalities as avoidable, unfair and inequitable, challenging the individual risk factor approach and medical solutions dominating much of 20^th ^century research. Developments include using the concept of embodiment to understand health outcomes as the manifestation of social inequalities [2] and the re-emergence of life course approaches investigating long-term and cumulative health effects of exposures [3]. Theoretical schemas combine ecological and social perspectives, including the eco-social model of Krieger [2], the eco-epidemiological model of Susser [4], and the social-ecologic systems perspective of McMichael [5]. The earlier social production perspective, which in Doyal with Pennell's [6] classic text interrogated relationships between commodity production, profit, and health, is explicitly included in Krieger's models. We intend this article to fit with these approaches, building on them by analysing specific social processes (capital accumulation and motorisation) and social forms (commodities and corporations) absent from most epidemiological work. We suggest how this approach can progress key debates on the causes of health inequalities and refocus attention from those at the bottom to the social processes underlying the distribution.

Traditional epidemiological tools have limitations for understanding social and economic determinants of health [5,7]. Social processes are not fully explained through the search for discrete effects after adjustment for confounding variables. While Rose's dictum that the determinants of health at a population level may be different from those at individual level has been influential in social epidemiology [8], reliance on analysis of variation within a population continues. Focusing on the existing distribution misses those etiological factors that vary little within a population, such as car-based transport systems, and the interests, often economic, driving the development of these systems. Excluded from the analysis, social factors are often relegated to the outer boxes of diagrams used to illustrate social determinants of health. Typically such diagrams are focused on the individual not the population, while catch-all terms such as "economic context" leave social processes unexplained and do not tell us how to operationalise them [7,9].

### Transport: a broader view

#### Starting with social processes

In this article we put forward an approach that starts from social processes and social forms (rather than inequality in an outcome or exposure) and moves on to analyse effects within specified social relationships. This can unpack the black box of "social context". We examine two commodities (the car and oil), their social and economic role, including the corporations dominating their production, how they contribute to patterns of health and illness, and obstacles public health professionals may face tackling these.

Similar approaches are used in tobacco control. Although smoking cessation is a core individual health promotion message, a broader critique analyses and responds to tobacco industry power. The health community has targeted tobacco as a commodity and the industry profiting from it, rejecting technical fixes such as extractor fans and "mild" cigarettes. Some authors have highlighted how changes in tobacco consumption were rooted in changes in tobacco production and the changing needs of the tobacco industry [10]. The tobacco industry has cast smoking as an individual choice by informed consumers, but researchers recognise the role advertising plays in creating addiction and socially legitimising smoking [e.g. [11]].

The health effects of the social process "globalisation" and economic neo-liberalisation have also been held up to scrutiny [12]. The globalisation literature has linked social processes to health outcomes (including the globalisation of the cigarette industry and its targeting of low income countries, producing a growing and unequal disease burden). Responding to the obesity epidemic, the health community is drawing attention to the increasingly concentrated power of agribusiness and supermarkets [13], explaining how individual choices are shaped within obesogenic environments [14]. However, these approaches remain unusual within epidemiology as a whole.

Transport has received relatively little attention within social epidemiology, with notable exceptions [15]. Public health researchers recognise the importance of walking and cycling, but these are often seen as leisure activities. While the recent European WHO report on the socioeconomic determinants of diet and physical activity for adolescents [16] discusses walking and cycling, it makes no reference to the car and how motorisation inhibits active travel.

In the UK literature, the car predominantly appears as a proxy for income within regression modelling. In one major health inequalities collection [17] transport is absent from the index, while "car ownership" is cited 35 times as an indicator of material well-being or small area deprivation. This reduces transport to the possession or absence of a car. By contrast, our social process approach considers health implications of the car not just as an object in itself or a marker for material (dis)advantage, but within the growing global system of corporate commodity production. The global passenger motor vehicle fleet could soon reach one billion and road transport already produces 17% of energy related carbon emissions [18]. If global ownership reached the US average (which is still increasing) there would be over five billion motor vehicles, an environmentally terrifying prospect.

A myriad of pathways link car-based, energy intensive transport systems to public health [19]. The burning of the fossil fuel, oil, produces the kinetic energy that kills 1.2 million people and injures 50 million on the roads each year [20]. Urban air pollution from motor vehicles is responsible for hundreds of thousands of deaths per year [21], while noise pollution is implicated in cardiovascular disease, and inhibits cognitive development [22]. The obesity epidemic is linked to the shift from human powered to fossil fuel powered transportation, which contributes to an imbalance between energy expenditure and energy consumption. Major roads can sever communities by barring local access, communication and social integration, with implications for social capital. These harms and benefits are unequally distributed. Researchers have identified socio-economic inequalities in road traffic injuries [23], obesity [14], exposure to air pollution [24], and health effects of urban sprawl and community severance [25].

Our theoretical approach responds to this interlinked complexity by foregrounding epidemiological implications of the car's economic and social importance to contemporary capitalist societies. It draws upon Marx's distinction between exchange value and use value [26], incorporating car cultures and car advertising through the concept of sign value [27]. Use value represents an object's instrumental function, exchange value its market price, and sign value the status power it confers to differentiate its owner from others. All of these are intertwined, with implications for public health and epidemiology.

#### Exchange value: car production

Exchange value directs us to the car's pivotal role within capital accumulation, which matters for health in three key ways. Firstly, it suggests the existence of key 'anti-health forces', corporations which depend for their survival on the maintenance and growth of car-based transportation systems. Secondly, transport consumes around half of world oil production [28], and oil-producing countries often suffer from the 'curse of natural resources'. Thirdly, the huge levels of investment allocated to car production and associated activities imply huge opportunity costs (including health costs) stemming from missed alternative resource uses.

##### The car and the world economy

Transport of goods and persons has long been economically important (as an economic sector in itself and by facilitating production and exchange in other areas). In modern capitalist society transport has developed in tandem with the corporate form, shaping the position transport and corporations hold within today's global economic system. The first corporations were formed to finance British and Dutch colonial shipping ventures. After scandals, corporations were banned in England in 1720; in 1776 the economist Adam Smith called them a recipe for negligence and profusion. Yet soon afterwards, the growing rail industry's need for large amounts of investment resuscitated the corporate form, and it became widespread [29]. Corporations now rival governments as the key social form through which society allocates productive resources. Importantly, this resource allocation does not emerge from Adam Smith's balance of small producers and consumers, but is directed by powerful organisations that co-operate within and across industries, and that lobby regulators, advertise, and strategise.

In the twentieth century, cars became central to the accumulation strategies of the corporate-dominated global economy. This has applied both to the post-war Keynesian Bretton Woods consensus and the subsequent neo-liberal orthodoxy, as is illustrated by looking at the top global corporations in 1970 and 2006 [Tables 1 and 2]. In 2006, nine out of ten top corporations by sales were primarily involved in selling cars or oil, compared to seven out of ten in 1970. Manufacturing car industry products "uses up nearly half the world's annual output of rubber, 25% of its glass and 15% of its steel. No wonder the car industry accounts for about 10% of GDP in rich countries [30]." The car economy supports associated industries (e.g. advertising, glass, production, finance, and insurance) and enabling industries (e.g. road building and maintenance).

**Table 1:** Fortune 500 top 10 corporations 2006 [97]

**Rank**	**Company**	**Revenues ($ millions)**	**Key product**
1	Exxon Mobil	339,938	Oil
2	Wal-Mart Stores	315,654	Supermarket
3	Royal Dutch Shell	306,731	Oil
4	BP	267,600	Oil
5	General Motors	192,604	Cars
6	Chevron	189,481	Cars
7	DaimlerChrysler	186,106	Cars
8	Toyota Motor	185,805	Cars
9	Ford Motor	177,210	Cars
10	ConocoPhillips	166,683	Oil

**Table 2:** Fortune 500 top 10 corporations 1970 [97]

**Rank**	**Company**	**Revenues ($ millions)**	**Key product (in 1970)**
1	General Motors	24,295	Cars
2	Exxon Mobil	14,930	Oil
3	Ford Motor	14,756	Cars
4	General Electric	8,448	Computers
5	IBM	7,197	Computers
6	Chrysler	7,052	Cars
7	Mobil	6,621	Oil
8	Texaco	5,868	Oil
9	ITT Industries	5,475	Telecommunications
10	Gulf Oil	4,953	Oil

The other corporation in 2006's top ten – Wal-Mart – is intimately tied into the car economy. Its model is based upon economies of scale that only exist with subsidised road transport, sprawled developments, and heavy levels of private car use. "Typical Wal-Mart stores generate several thousand average daily car trips" with supercentres generating far more [31]. Truck-based freight systems and car based passenger transport have grown together and reinforced each other. Initially, transport corporations developed to move freight, but from the middle of the 19^th ^century passenger rail took off, and at the end of the 19^th ^century, personal vehicles – first bicycles and then cars – pioneered mass production processes. Since then, truck and car interests have been closely intertwined.

##### Vested interests

Corporations that produce cars, oil or other associated commodities have vested interests in maintaining and increasing sales of these commodities. They have massive fixed capital invested in refineries, wells and factories and an established position within the global economy, with the economic and political benefits this offers. Car firms act to maintain this [32] through advertising, lobbying, and the promotion of technical fixes and individualised solutions. Budgets for car advertising tower over budgets promoting walking and cycling. Transport for London, an authority with a notable pro-cycling stance, expects to spend only £600,000 per year on cycle mapping *and *promoting cycling throughout the Greater London area [33]. This means occasional bus stop advertisements, by contrast with billions spent on car related advertising, which buys giant 48- and 96-sheet billboard posters and ubiquitous television and internet presence. Although around a quarter of trips in the United Kingdom are on foot, pedestrians have little power as a lobbying group.

While growing car dominance has rarely been seriously challenged, the industry has had to respond to climate change. In 1989, major car firms were founding members of the Global Climate Coalition, a group of powerful United States businesses opposing environmental regulations, which achieved its aim of pulling the US out of the Kyoto treaty. The UK's Royal Society (national academy of science) recently wrote to oil giant Exxon expressing "concerns about the support that ExxonMobil has been giving to organisations that have been misinforming the public about the science of climate change [34]." Other oil and car firms now adopt publicity strategies which claim to support the environmental agenda, yet Shell's investment in renewables in 2005 was 1% of total capital investment, compared with 69% spent on searching for fossil fuel fields [35]. Recently, car lobbyists helped persuade the European Commission to relax proposed emissions regulations [36]. In a recent Friends of the Earth study, 96% of ads in UK newspapers were for cars breaking the current voluntary emissions limit [37]. Meanwhile, the US Alliance of Automobile Manufacturers set up the well-funded DriveCongress campaign [38] to oppose a bill agreed by the US Senate to increase fuel economy standards on new vehicles to 35 miles per gallon by 2020.

Corporations and related groups tied to the car also try and shape approaches to road safety through organisations such as the Global Road Safety Partnership, the Fédération Internationale de l'Automobile (FIA), and the Commission for Global Road Safety [39]. Promoting education over regulation has been a favoured industry strategy for alcohol, tobacco, and road safety [40-42]. This is despite evidence that it is less effective than interventions aimed at restricting advertising, directly reducing access and use, and increasing price [43,44]. Road lobby interests are well-funded and given the process of motorisation are often no longer recognised as vested interests. In the UK long established car rescue organisations, which profit from increasing motorisation, are widely consulted on transport issues while lobbying for motor industry interests.

##### The curse of black gold

The car economy creates oil dependency in both producing and consuming countries. In 1912, Churchill switched the British Navy from Welsh coal to Persian Oil [45], since when it has been a military imperative to ensure continued oil supplies even during war. The expansion of oil-based road transport has further locked rich Western economies into oil dependence. Impacts on producers have been principally negative; they tend to have poor health and development [46] with oil export dependence linked to child malnutrition, low healthcare spending, low school enrolment, and poor adult literacy. One study found that each 5% rise in oil exports to Gross Domestic Product (GDP) reduced life expectancy by four months and increased malnutrition by 1%. [47]

Competing explanations exist, but recent research has noted oil producers' increased risk of corruption, often fuelled by Western corporations, authoritarian government, poor governance, high military spending, and civil war [48]. This is exemplified by the catastrophe in Iraq, one of the few countries where road traffic crashes are not the most common cause of violent death. The war may not be reduced to oil but they cannot be divorced, as many commentators have noted, including former head of the US federal reserve Alan Greenspan [49]. A 2007 survey estimated 1.2 million people in Iraq had died in conflict following the 2003 invasion [50]. Connections between these deaths, oil dependent states' underdevelopment, and the world's largest corporations are of major epidemiological significance.

##### Opportunity costs

Car dependent transport systems are resource intensive, in particular energy intensive [19], and facilitate other resource intensive patterns of development, including deforestation. As the most expensive commodity purchased by most households (housing costs primarily represent interest and rent not construction costs) the car has helped to ensure the growing consumer demand required for capital accumulation. Gorz and Illich [51,52] have described car economies as locked into radical dependency, where massive resources are required for tasks (e.g. household shopping) that could be completed using far fewer resources, on foot or by bike. This creates a huge opportunity cost both to the individual and society. If these resources were devoted to primary health care, sanitation or providing electricity to the world's energy poor the health gains could be enormous.

##### Use value: car consumption

While the concept of exchange value directs attention to production processes, use value refers to consumption or how cars are used by individuals to fulfil needs and desires. These are socially shaped, just as their resolution shapes societies and environments. We do not only consume "things", but "things which are part of a definite social environment [53]." This matters for epidemiology, as it directs our attention to the health effects resulting from this interplay between social structures, individual decisions, and commodities.

Socially produced, individually purchased, and consumed in a social context, use values affect people other than the purchaser. In neo-classical economics these consequences are conceptualised as (positive or negative) externalities, a useful concept, although one too narrowly conceived by such economists, who see the externality as the exception [54] and believe that monetisation can solve problems of health and social justice. Use values and accompanying externalities incorporate health effects, and the concept of use value directs our attention to the social context of consumption. A society using automobiles to provide a taxi service on a needs basis would produce different health effects to one using private cars as the primary method of mass transportation.

Within the latter system particular features of the car create a vicious circle driving out healthier and sustainable alternatives. Here, three aspects of this process are discussed. Firstly, we consider violence and the threat of violence. Secondly, we discuss the car and the restructuring of place. This environmental transformation enhances the car's use value to consumers with the creation of obesogenic and socially divisive environments. Finally, we suggest how "car cultures" enhance the car's sign value or ability to act as a marker of social differentiation. This has connections to social epidemiological work on the construction of unequal and hierarchical societies.

##### The car and violence

We propose that violence and the threat of violence – the threat of a physical force effecting serious injury – is part of the car's use value to consumers. Car violence is a consequence of the transformation of vast amounts of fossil fuel energy into kinetic energy. The threat of violence is present irrespective of the driver's intentions and is built into roads designed for speed, but its intentional use is audible in the soundscape of motorised societies: engines revving and horns honking at slow pedestrians.

Motorised violence is socially naturalised, and justice systems in many countries impose minimal penalties upon drivers causing death or serious injury. The term "accident" (an unforeseen event or one without an apparent cause) is used to describe the predictable effects of high-speed motor vehicles. "Car crime" invokes crimes against cars, while the softer term "driving offence" covers the exercise of lethal force. This evasion is reflected in some health inequalities literature. Wilkinson [55] extensively discusses poor-on-poor violence ignoring motorised violence (more typically rich-on-poor violence). Yet his stress on the real lack of respect shown to the poor is exemplified in the treatment of different road users. While the psycho-social literature has focused on important inequalities in work related stress, the daily life and death decisions needed to navigate traffic and the chronic stress from noise pollution are less frequently considered.

Road traffic crashes remain a neglected epidemic, despite being the most common cause of violent injury in almost all countries [20]. These harms are naturalised as part of the development process although assumed benefits are questionable [19]. Crashes are inequitable, with most victims being pedestrians and cyclists who would never own cars. In addition to the human suffering, road crashes impose high economic costs on already poor countries. Surprisingly, the transport dimension is so far absent from much globalisation analysis; even that focused on the negative ways in which low income countries are integrated into the world economy.

The traditional road safety approach endorsed by the motor lobby tends to focus on the behaviour of the victims or a few errant drivers, rather than the source of danger. In response to road dangers, pedestrians and cyclists are instructed to give priority to motorised transport and keep out of its way. According to Roberts "[r]oad user education ... sends the message that road space belongs to drivers, and that pedestrians and cyclists must look out or die [40]." Although they pose minimal threat to others, walking and cycling are seen as dangerous choices to be regulated through helmets and controlling "jay walking".

Analysts' choice of health indicator can obscure complex car-related processes leading to diverse health effects. The injury rate per motor vehicle kilometre, which typically falls as motorisation increases, hides declining active transport. Indicators such as the injury rate per kilometre walked or cycled, or per hour playing in the streets, would be better starting points for understanding how environments hostile to active transport are being created and how health professionals might effectively counter this.

If motorised violence is embodied in road injuries and deaths, the threat of violence is embodied through obesity, diabetes, and cardiovascular disease. The reduction in active travel is fuelling the obesity epidemic [56] and reducing the social participation of already disadvantaged groups: children, older people and those with disabilities [57]. Harms from physical inactivity are not equally distributed. Although lower income groups typically have higher levels of transport physical activity for work and shopping, they have less opportunity to participate in leisure related physical activity [58] and are more vulnerable to passive obesity [14]. Lower income groups in inner-city areas are often separated from the countryside by affluent suburbs, and exposed to high levels of air pollution despite their low car use [24].

##### The car and place

Studies often use multilevel modelling to investigate if "area effects" explain geographical differences in health over and above the composition of people living there. From this it is argued that "if variations in health between areas can be entirely explained by characteristics of the inhabitants of those areas, policymakers need only act on improving the circumstances of individuals [59]." This approach has been criticised for the lack of theoretical justification for selecting areas, the ahistorical analysis and for not considering the constitutive relationship between person and place [7,60,61]. We suggest our focus on social processes facilitates consideration of this constitutive relationship.

Area and individual characteristics are not static, separate "things", but related moments in historical and spatial processes. People experience what is apparently 'the same area' differently. The same house may have different properties and health effects for the car driver and for the child who cannot cross the congested street, while a frequent flyer's neighbourhood may include global airports but not the nearby council estate. A neighbourhood or community is not a fixed area but relative to an inhabitant's mobility. As Freund and Martin [62] argue, "Sociomaterial space is not simply inert material--an infrastructure of asphalt and concrete--but expresses and structures social life. Social space is space that is used ... we need to replace a static conception of space with a 'movement based' one. In movement, time and space are inextricably linked."

We see the car's ability to annihilate distance as a key use value for the consumer. Car manufacturers benefit from the creation of such distances, because mobility becomes more useful as distances grow. In this way, mass car ownership, backed by corporate lobbying for pro-car policies, builds car-dependency into the urban fabric [63].

Firstly, accommodating environments to the car encourages social segregation. Cars require copious space for parking and driving; these needs can be met through zoning and/or geographical social segregation, often around ethnicity or class. Social segregation physically embeds unequal social relations into the built environment, strengthening these divisions. In the UK " [i]ncreasing access to cars over the past three decades has allowed the population who can to segregate further [64]."

Secondly, transport engineering based around the car creates distance for non-car users. Traffic volumes and road design make other road users' journeys slower or less direct, and it becomes more expensive and less energy efficient to run a frequent public transport system. The main road that destroys distance for the driver can simultaneously block access to local amenities for the pedestrian and cyclist. Thirdly, amenities literally move away from people, through a chain of events that leads to a lack of local shops, services, and jobs ("the death of the high street": Simms [65].)

Together, the threat of violence and the restructuring of place raise the bar for social participation, essentially disabling large segments of the population [57]. In particular, the independent movement of children has been curtailed [66]. As Jain and Guiver [67] comment, "When viewed from the perspective of society as a whole, rather than the individual car user, the move towards greater mobility can be largely seen as self-defeating... [resulting in] increasing dependence upon motorized transport." Motorisation creates distance only the car can overcome, with cross-sectional analysis finding a strongly inverse relationship between accessibility and motorisation [68].

Sprawl and its impact on communities has been linked with ill-health, through both material and psychosocial pathways [69]. Land used for roads or driveways cannot be used for agriculture or forests and increasing the built up surfaces harms drainage and water quality [69]. Sprawl is seen as weakening neighbourhood ties reducing social capital. This insight needs to be linked to how economic capital is accumulated and laid down in places; as car-dependent suburbs, car parks, roads, refineries, and factories [70]. This foregrounds the relationship between the car and the profitability of alternative accumulation strategies. Economic capital on balance sheets represents social relations, both between competing owners of capital and those without capital. Effects of these power asymmetries can be subtle or overt, as when Barcelona city authorities re-started major road building programmes in the 1980s, after car firms threatened to leave the city and so destroy thousands of jobs [71].

These processes have been contested. Cities like Bogotá and Copenhagen have made positive steps to retain or develop public spaces for people rather than motor vehicles. Although local political organisation can make a difference, city-based responses are undermined, as economic forces produce leapfrogging development and policy at higher levels continues to promote mobility over accessibility [72].

##### Sign value and car cultures

Finally, sign value directs our attention to the car's cultural and psycho-social role, in particular to advertising. The car is the most advertised commodity [73], and while capital has become increasingly concentrated in the hands of a small number of car firms, car *brands *have proliferated [74]. Although sociologists have analysed "car cultures" [75], negative health and environmental consequences of mass car ownership are frequently separated from this cultural analysis. But just as we would criticise those who abstract health from social relationships, we believe that it is mistaken to consider cultural forms abstracted from their health and environmental aspects.

We would argue that car ownership is connected to an increasingly toxic system of sign values, which feeds off and encourages the threat of violence described above. Firms promote vehicles as aggressively conquering Nature or urban space, with the recent Lexus LX470 campaign even stating "Now with added intimidation" [76]. Environments are depicted as hostile places from which cars provide protection. Vehicles are sold within a militarised context as "urban proof" and "rock hard" (the Nissan Navara, promoted in conjunction with the film Die Hard 4.0). As "urban" carries racialised connotations [77], such imagery and language may reinforce racism. This advertising genre creates and builds upon hierarchy and anxiety, feeding an "ecology of fear" [78] in which "bad dreams" define and control public landscapes.

Even where car advertisements do not glorify aggression, they frequently promise speed and control over the external environment [74], which may contribute to stress and road rage when drivers experience the conflict between potential speed and congested reality. Some authors have called the car a positional good, one that provides benefits where others have less of them. This aspect of the car's (dys)functionality adds to the discord between individual rational choices and societal ones [79].

Car advertising could have psycho-social effects upon health both directly (through encouraging the purchase of larger and more dangerous cars) and indirectly (through the validation of 'macho' behaviour when driving and more generally). Furthermore, ubiquitous advertising helps to normalise sloth. Driving has become such a normal part of everyday life [75] that many car owners no longer question using cars for short journeys [56]. Conversely, walking ceases to be a normal activity and becomes a type of "exercise" that health agencies struggle to promote.

## Discussion

The car economy matters for health and for epidemiology. Linking together injuries, obesity, air pollution, conflict and climate change with the social processes that have established car dominance offers a new direction for social epidemiology. It shows how attempts to overcome distributional inequalities by encouraging the poor to emulate the motor vehicle use levels of the rich would prove catastrophic in the context of climate change.

### Eco-social epidemiology

Most social epidemiological theories incorporate ecological thinking [4,5]. A key ecological concept is metabolism. Transport is central to our individual and social metabolism, shaping our relations with each other and our relation with nature and transformed nature, including our extractive relationship with the environment. Our maladjusted metabolism can be seen acting on individual energy imbalance (through too much food and fossil fuel-powered transport) leading to obesity [80], and on climatic destabilisation through increased atmospheric carbon concentrations. We need new epidemiological thinking about these linkages and the economic system that produces them.

### Moving beyond the material versus psychosocial debate

Focusing on economic and social processes helps move beyond the debate over material versus psychosocial explanations for health inequalities. Psychosocial theorists have provided genuinely social theories in attempting to explain the distribution of health outcomes by reference to socio-economic structures, and innovatively combine sociological and epidemiological approaches. Marmot et al [81] point to lack of control over one's work as an important factor causing socio-economic gradients in health, which cannot be explained by absolute poverty. Wilkinson [55,82] has argued that socio-economic structures affect health directly through social-psychological processes.

Neo-materialist critics claim that psycho-social theories introduce unnecessary and ahistorical meta-explanations to explain diverse and contingent pathways [83]. They focus upon cause specific, material explanations, arguing that instead of psychological states influencing health directly, "misery is now a marker for material disadvantage [84]." Neo-materialists argue that psychosocial theories lead to problematic policy effects, focusing on poor people's mental state rather than their poverty [84]. Yet focusing on material disadvantage can be as restrictive as focusing on its psychological effects.

As Whitehead [85] argues, while more amenable to policy makers, targeting the poor is less likely to be effective than population-based interventions. The effects of car dependency clearly illustrate this. Firstly, automobile-related disease, injury, and deaths are disproportionately caused by the rich and suffered by the poor [24], so targeted interventions might more effectively – and fairly – be aimed at reducing the harmful behaviour of the former. Secondly, a focus upon equalising the distribution of harms at the expense of addressing broader issues of harm causation implies assisting people to fit into a car dependent system that is unsustainable and inequitable. As with cigarettes and energy-dense food, increased consumption of cars would not be beneficial for health. Targeting the powerless can obscure the importance of addressing harmful actions by the powerful (corporations and individuals) and the urgent need to address broader systemic issues.

The material and psychosocial should form part of a joint social epidemiological narrative. The material is always experienced psychosocially. A focus on social processes offers insights into the material basis of psychosocial pathways, such as the stress of navigating dangerous traffic and the hostile attitudes encouraged by much car advertising. Our approach seeks to integrate the two within a more sophisticated, process-oriented, materialism that contests the automatic benefits from economic growth (expanded commodity production), without giving dominance to psychosocial pathways.

### Demotorising society

The WHO has defined health as a state of complete physical, mental and social wellbeing. An important question for epidemiologists should be what kind of society would promote this. We believe it would be one where physical activity is part of everyday life, with safe and attractive environments for socialising and playing, clean air, and equity in accessibility by age, ethnicity and sex. We think that this would mean car-free cities; urban transport based around walking and cycling would be the best modes for most people for most journeys, public transport would be used for longer journeys, with small, light electric vehicles for carrying heavier loads and when people are unable to walk or cycle [86]. A recent study found that only a car-free London could achieve target greenhouse gas emission reductions (60% by 2030) [19]. We argue elsewhere that active travel, in particular cycling, can be more inclusive than is commonly recognised [57].

Social epidemiological approaches rightly caution against technical or pharmacological fixes for socially caused problems, and indeed the "eco-car" is a fantasy [86]. However, we do not argue that the car as an object is bad in itself; instead objects must be understood within the social processes that surround them. The situation in which cars are sold as commodities within societies increasingly built around the presumption of mass car ownership can only exacerbate harms – and ensure that the richest continue to enjoy the lion's share of any benefits.

### Research operationalisation

We would not argue for prioritising one particular technique: there is not one right way to do social epidemiology. However, suggestions for research would include the under-explored areas of car advertising, the motor industry and road safety initiatives, and international grants and loans for road building. More broadly, we call for an approach centring the role of the car in socio-spatial processes leading to socioeconomic and geographic health inequalities. Terms in the critical transport literature such as car dependency [87,88] should be operationalised within quantitative and qualitative epidemiological analysis. Car dependency has been used to analyse how car-based societies disable non-car users [57]. This has implications for health metrics, in particular Disability Adjusted Life Years (DALYs), which require consistency in disadvantage of a given health state. A physical impairment may not be experienced as a disability in an accommodating society, while in a car-dependent society the mere lack of a car can be considered a disability.

Recently-developed epidemiological techniques could fit within our general approach; for example Health Impact Assessment (HIA) allows an integrated approach to the effects of policy changes, and requires explicit description of the proposed pathways [89]. While typically HIA has been constrained by policy debates, it can be used for public health activism.

Comparative risk assessment (CRA) has compared radically different alternative scenarios for the Global Burden of Disease report [90]. This approach fits with Rose's call to find the causes of incidence rather than focusing on relative risks [8]. However, the inclusion of both commodities and behaviours in the report and their isolation from the social processes that connect them limited the impact of this project. Road traffic injuries were considered as a burden of disease by cause, while physical inactivity, overweight and obesity and urban air pollution were all separately presented in the risk factor assessment. Integrated assessment of the health impact of alternative transport scenarios would link these pathways [91].

Scenario-based approaches are most developed in the study of climate change [92], where multiple and complex pathways require new techniques [5]. These approaches have highlighted the environmental and health consequences of different social trajectories. From here, it is important to take the next step and investigate structures and organisations that act as barriers to achievement of better population health.

### Identifying anti-health forces

The UK Public Health Association calls for a focus on challenging anti health forces [93]. Although the relation to health is simpler, we think much can be learnt from how health professionals have approached tobacco. There is recognition that the tobacco companies are anti-health forces and cigarettes are a commodity to be challenged. Yet initially the tobacco industry had been consulted as a major employer and industry.

Corporations have become the subject of revived political critique and climate change campaigners have calculated the greenhouse gas emissions of corporations [94]. Similar calculations could be made for deaths from road crashes or oil-related air pollution. As Lynch et al. point out, reducing population-wide risk factors is required and " [u]ltimately this will mean engaging the political and economic forces that have interests in maintaining profits from the sale of products and services that influence conventional CHD risk factors [95]." However, in practice, engagement is on-going. The question is the form it should take. We suggest oil and car corporations are anti-health forces, and collaboration with them creates fundamental conflicts of interest.

We would see partners as communities that suffer most, particularly where campaigns already exist, such as the global People's Health Movement, and the environmental justice movement in the United States.

## Conclusion

The particular accumulation strategies followed by the world's largest corporations are closely linked to two intertwined commodities, the car and oil. These strategies and the processes of motorisation that have accompanied them act upon health through injuries, obesity, conflict, air pollution, and climate change. Understanding these linkages offers the chance to develop a more nuanced materialism that incorporates but does not start from psychological pathways, which can help explain why greater average material consumption is not automatically associated with improved health. Moreover, it requires us to reconsider the main barriers to achieving environmental sustainability and improving population health. As Rose argued [96] "*A radical approach aims to remove the underlying impediments to healthier behaviour, or to control the adverse pressures. The first or medical approach is important, but only the social and political approach confronts the root causes*."

## Competing interests

The author(s) declare that they have no competing interests.

## Authors' contributions

JW: wrote and edited the article. RA: co-wrote and edited the article.
